# Predictors of adolescent well-being around the globe: are students from Confucian East Asia different?

**DOI:** 10.3389/fpsyg.2024.1446301

**Published:** 2024-10-28

**Authors:** Robert Rudolf

**Affiliations:** College of International Studies, Korea University, Seoul, Republic of Korea

**Keywords:** subjective well-being, adolescents, culture, peer well-being, Confucian Asia, collectivism

## Abstract

**Purpose:**

Using rich data from nearly 400,000 15-year-olds across 70 middle- and high-income countries and economies participating in PISA 2018, this study investigates (1) global predictors of adolescent subjective well-being (SWB), and (2) differences in adolescent life satisfaction, its predictors and endowments with predictors across world regions and cultures. A particular focus lies on comparing Confucian East Asia (CEA) with other world regions.

**Methods:**

Data were analyzed using multiple linear regressions and Blinder-Oaxaca decomposition. As measures of adolescent well-being, this study employs life satisfaction, affective well-being, and meaning in life.

**Results:**

Globally, adolescent well-being outcomes are found to be most strongly linked to gender, personality, relative SES, relationship quality, peer SWB, autonomy and the learning environment, as well as local cultural factors. Estimations by world region reveal several culture-specific explanations for interregional well-being gaps. In particular, notoriously low levels of life satisfaction among students from CEA countries are found to be associated with low self-efficacy, low peer well-being, as well as with high emotional interdependence compared to other world regions. Emotional interdependence is more strongly experienced among CEA adolescents compared to adolescents from any other world region. Moreover, it is found to be more strongly associated with life satisfaction in the CEA region than in any other region. In line with the former, CEA students show stronger links between other relational factors (parents’ emotional support; sense of belonging at school) and life satisfaction compared to most other regions.

**Implications:**

This study suggests that among the environmental factors that shape the experience of adolescent lives, relationship and cultural factors play key roles and are closely intertwined. Parents, educators and policymakers around the world should focus on creating a positive school environment that promotes well-being, student self-efficacy, a sense of belonging, and a safe space in which failure is accepted as part of the learning process. This is particularly needed in Confucian East Asian countries.

## 1 Introduction

Over the past two decades, subjective well-being (SWB) measures have increasingly been used in the study of child and youth well-being. Following Diener (1994), SWB refers to individuals’ evaluation of the quality of their lives in general. It is a multidimensional construct that includes affective and cognitive components. In particular, it is cognitive component—overall satisfaction with one’s life—has become a key comprehensive measure of adolescent well-being across countries and cultures (Diener and Diener, 1995; Gilman and Huebner, 2003, 2006; Antaramian et al., 2008; Bradshaw et al., 2013; Lee and Yoo, 2015). Besides being important in its own right, adolescent SWB is known to contribute to a variety of positive youth outcomes while buffering against negative ones. Low life satisfaction in adolescence has been found to be associated with psychological, social and behavioral problems. In contrast, high life satisfaction has proven to mitigate the negative effects of stressful life events, to reduce participation in risky behaviors, and to be conducive to good mental health (Park, 2004; Suldo and Huebner, 2004; Mahmoud et al., 2012; Sun and Shek, 2013). Life satisfaction has further been found to be positively correlated with a variety of desirable psychological characteristics (Park, 2004). Within the school environment, students with higher life satisfaction have shown stronger cognitive engagement and have been found to be more resilient to school-related stress (Lewis et al., 2011; Moksnes et al., 2016; Shek and Li, 2016). Adolescent SWB further matters for the transition to adulthood and many long-run life outcomes including adult life satisfaction (Fredrickson, 2001; Coffey et al., 2015). Related adult studies further suggest that happier people are more likely to get married (Stutzer and Frey, 2006), may be more successful on the job (Zelenski et al., 2008), and enjoy healthier and longer lives (Diener and Chan, 2011).

Studies among children and youth have consistently found that demographic and socio-economic variables can only explain a small portion of the variation in life satisfaction (Gilman and Huebner, 2003, 2006). Indeed, most of its variance can be ascribed to intrapersonal and interpersonal environmental variables (Levin and Currie, 2010; Morgan et al., 2012; Lee and Yoo, 2015; Coupe and Obrizan, 2018). First, similar to adults, the largest fraction of life satisfaction differences across adolescents can be explained by intrapersonal differences, i.e., differences in personality traits (e.g., extraversion, neuroticism, locus of control, self-esteem, self-efficacy, independent vs. interdependent) (Diener and Lucas, 1999; Park, 2004; Steel et al., 2008; Li et al., 2019). Second, interpersonal environmental factors such as relations with family and friends account for another large fraction. Among school-related factors, peer relationships, a sense of belonging, and academic stress have been found to be associated with student well-being (Lee and Yoo, 2015; Chiu et al., 2016; Lemma et al., 2015; Moksnes et al., 2016). Generally, research suggests that adolescent life satisfaction can be enhanced by supportive parenting, engagement in challenging activities, positive life events, and high-quality interactions with significant others.

Since Diener and Diener (1995) found substantial cross-country differences in subjective well-being, many studies have attempted to contribute to the explanation of such differences. Early studies showed cross-country SWB to correlate strongly with countries’ levels of income (e.g., Diener and Biswas-Diener, 2002). However, even after controlling for material wealth and other macro factors, substantial SWB differences remain across countries and world regions (e.g., Deaton, 2008; Inglehart et al., 2008; Senik, 2014; Helliwell et al., 2018; Rudolf, 2020). In particular, given their income levels, average reported SWB among members of Confucian East Asian countries such as Japan, Korea, and China has been found to be notoriously low, while that of most Latin American countries was found to be surprisingly high. Studies further have shown that cross-country differences in SWB are not just an artifact of language issues or varying levels of modesty in interview situations, but are correlated with related measures of mental health such as vitality and the absence of depressive symptoms (Senik, 2014; Casas and Rees, 2015; Chen et al., 2015). Therefore, it is now widely acknowledged that *cultural factors*—the norms, values, and practices shared by a group of people—play a key role in explaining SWB differences across countries. Social values, norms, and believes set the boundaries in which emotions are permitted and expressed in a given group, thereby framing emotional experience (Fulmer et al., 2010). Culture has further been found to influence how much *importance* people place on various aspects of their lives when making life satisfaction judgments. Most studies in this field have focused on comparisons between Americans and (Confucian) East Asians^1^, often using university student samples, and have employed Hofstede’s classification of cultures into individualistic and collectivistic (Diener and Suh, 2000; Park and Huebner, 2005; Oishi and Diener, 2009; Suh and Choi, 2018). In these studies, people from Western/individualistic cultures have been found to weigh self-esteem and emotions more when making life satisfaction judgments, while members of Confucian East Asian/collectivist cultures have shown to place greater importance on relational factors, particularly on family and friends (Diener and Diener, 1995; Suh et al., 1998; Park and Huebner, 2005; Uchida and Ogihara, 2012). Suh and Choi (2018), in a recent review of the literature, posit that “East Asians’ excessive concern of other’s view might be a key psychological reason for why their happiness level is lower than expected by economic indices.”

In a recent study, Rudolf and Bethmann (2023) use PISA 2018 data from 72 middle- and high-income countries to show that *adolescent* SWB is negatively related with a country’s per-capita GDP, the opposite of what can be found for *adult* data. The authors point to higher learning intensity and school-related stress in advanced countries as a potential mechanism that drives this apparent paradox. Focusing on the case of South Korea, and also using PISA 2018 data, Rudolf and Lee (2023) show that a more competitive school climate induces higher academic performance in mathematics, reading, and science. Student competition, however, comes at the cost of reduced individual life satisfaction. Stankov (2010) shows that high academic achievement of students from Confucian Asian countries is accompanied by high levels of anxiety and self-doubt. He argues that an “unforgiving” Confucian culture, coupled with the belief that effort rather than ability is the primary source of success, might be causing this phenomenon. Fwu et al. (2017), using data from Taiwan, argue that in a Confucian cultural context, students are trapped in a dilemma between “feeling bad” (emotional distress) for exerting excessive effort and “being bad” (negative view of others) for making little effort.

Cultures can also differ in their desirability judgments about happiness. Western cultures often hold that happiness is an inalienable right and a product of an individual’s action. On the contrary, such belief is less prevalent in East Asian or Islamic cultures (Joshanloo et al., 2014). Bastian et al. (2014) show that in cultures where positive emotions are highly valued, people report higher life satisfaction and report more positive emotions. At the same time, collectivist East Asian cultures are currently undergoing a gradual shift away from collectivism and toward individualism (Uchida and Ogihara, 2012).

Additional contributions on the cultural making of well-being have recently come from self-determination theory (SDT). According to SDT, individuals reach higher levels of well-being when satisfying their basic psychological needs of autonomy, relatedness, and competence (Ryan and Deci, 2000, 2017). Conzo et al. (2017) provide an empirical test of SDT and find for 27 European countries that lack of trust, high obedience, and low respect constrain the satisfaction of basic psychological needs, and thus in turn lower individual well-being. Chen et al. (2015) apply SDT to a sample of university students across four countries with different cultural backgrounds (USA, China, Peru, and Belgium). The authors show that Chinese students score lowest in self-reported autonomy and relatedness, and report lowest vitality and life satisfaction. On the contrary, Peruvian students score highest in their satisfaction with all three psychological needs, and report highest vitality and life satisfaction, as well as lowest depressive symptoms. The study finds a strong statistical association between psychological need satisfaction and life satisfaction. Among all three needs, it shows that autonomy satisfaction is the strongest predictor of life satisfaction.

Past studies on the cultural making of life satisfaction have focused on a limited number of countries/cultures and have often been sparse in terms of explanatory variables included. In addition, given that most earlier studies have used university student samples, little is known about the cultural factors that explain *adolescent* well-being differences across countries/cultures. The present article aims to expand the understanding of adolescent well-being by using OECD’s PISA 2018 data for 70 countries from 10 major world regions.^2^ In particular, this study strives to (1) identify global predictors of adolescent subjective well-being; (2) examine cross-regional/cross-cultural differences in both endowments with predictors and in their relationship with adolescent life satisfaction; and (3) explain the gap in adolescent well-being between Confucian East Asia and other world regions.^3^

The present study contributes to the existing literature in the following ways. First, the present study uses one of the largest adolescent samples employed in the literature to date, both in terms of countries (70 from 10 world regions) and individuals (*N* = 398,609 15-year-old students). Moreover, while past studies have often relied on small-scale student samples, this study uses nationally representative student survey data. Second, this study focuses on the gap between Confucian East Asia and nine other world regions in the generation of well-being. Past studies have often only compared East Asians with one or two other cultural groups (often with European Americans), and there are only very few recent studies that have started using a larger number of countries and nationally representative survey data (Lee and Yoo, 2015; Marquez and Main, 2020; Rudolf and Bethmann, 2023). Third, this study makes use of a broad set of well-being outcomes and predictors. Life satisfaction, happiness, positive affect, negative affect, and meaning in life will be employed as outcome variables in order to provide a holistic picture of adolescent well-being. Moreover, predictors used include a comprehensive set of variables related to demographics, personality, socio-economic background, autonomy and home learning environment, peer characteristics, relationship quality, school atmosphere, and location. Lastly, as a methodological innovation, I employ a Blinder-Oaxaca decomposition technique in order to disentangle mean life satisfaction differences between Confucian East Asia and all other world regions into endowment and coefficient effects. In a nutshell, this technique allows disentangling region-specific differences in *endowments* that foster/constrain adolescent well-being from cultural differences in the *importance* that adolescents place on various aspects of their lives when making life satisfaction judgments. While commonly used in gender or racial gap analysis, Blinder-Oaxaca decomposition techniques have only recently been introduced to SWB analysis (Senik, 2014).

The remainder of this article is organized as follows. Section II introduces the data set, variables used in the analysis, and the empirical strategy. Section III presents estimation results by (1) examining global predictors of adolescent SWB, (2) conducting a region-specific analysis, and (3) decomposing mean life satisfaction differences between CEA and other world regions. Section IV summarizes and discusses the findings and concludes.

## 2 Data and methodology

### 2.1 Data set

This study uses data from the 2018 round of the Program of International Student Assessment (PISA). PISA is a triennial survey of 15-year-old students around the world that assesses knowledge and skills considered essential for full participation in social and economic life (OECD, 2019). PISA 2018 collected data from a total of 79 participating countries and economies, making it the largest cross-country student assessment to date. While the main focus of PISA is on conducting internationally comparable tests in reading, mathematics, and science, it also collects broad information on each participating student’s individual and family background, as well as teacher and school-related information. Some 600,000 students completed the assessment in 2018, representing about 32 million 15-year-olds in the schools of the 79 participating countries and economies.^4^ In 2018, comprehensive subjective well-being questions have been part of the PISA student questionnaires in 72 countries/economies, which allow for an in-depth analysis of adolescent well-being and its predictors across countries. This study uses data from up to 398,609 students from 70 countries/economies for which full information on all variables used in the analysis is available.

### 2.2 Variables

Following Diener (1994), subjective well-being refers to individuals’ evaluation of the quality of their lives in general. It is a multidimensional construct that includes affective and cognitive components. SWB traditionally consists of three components: relatively high levels of positive affect, relatively low levels of negative affect, and the overall satisfaction with one’s life. Researchers have often preferred measures of life satisfaction because these are more stable over time compared to affective measures which capture rather momentary feelings. Life satisfaction can be considered a comprehensive measure of adolescent psychological well-being (Park, 2004). In addition to these traditional SWB measures, meaning in life has received increasing attention lately as a measure of eudaimonic well-being (Steger et al., 2008; OECD, 2013; Yang et al., 2017).

Table S1 in the electronic supplementary file a detailed description of all variables and indexes used in this study. Five standard SWB outcomes (life satisfaction, happinessss, positive affect, negative affect, meaning in life) will be employed. A student’s life satisfaction is a single-item variable measured by asking students “Overall, how satisfied are you with your life as a whole these days?.” It has a response scale from “0” indicating “not at all satisfied” to “10” indicating “completely satisfied.” PISA 2018 also asked students about their usual positive and negative emotions. Student “happiness” is the response to the question “Thinking about yourself and how you normally feel: how often do you feel happy?,” where the response scale has four categories (“1” = “never,” “2” = “rarely,” “3” = “sometimes,” “4” = “always”). In the same fashion, the questionnaire asks students about other positive and negative emotions. Individual averages across all four positive emotions (happy, joyful, cheerful, lively) were used to construct a measure of “positive affect,” while averages across the four negative emotions (afraid, scared, sad, miserable) were used to construct a measure of “negative affect.” Lastly, meaning in life is measured using a 3-item index of perceived meaning in life.^5^,^6^

Several variables have been included to control for students’ individual characteristics. These include a female dummy, a single child dummy, a migration background dummy, as well as two personality-related measures. Several earlier studies have found girls to experience lower SWB than boys during their teen years, even in relatively gender-equal cultures (see for example Coupe and Obrizan, 2018). More importantly, personality traits play a key role in explaining interpersonal differences in subjective well-being (Diener and Lucas, 1999; Steel et al., 2008). This study therefore includes a 5-item index of self-efficacy/resilience and a single-item measure of emotional interdependence. Self-efficacy can be defined as the perceived ability “to deal with prospective situations” (Bandura, 1982) and has regularly be found to correlate with SWB (e.g., Tong and Song, 2004). I define emotional interdependence as the level of agreement to the statement “When I am failing, I worry about what others think of me.” The latter variable can be expected to play a larger role in collectivistic compared to individualistic cultures (Tsai and Lau, 2013).

Three variables control for family characteristics, i.e., parental education, an index of home possessions, and a 3-item index accounting for the level of parents’ emotional support perceived by the adolescent. Moreover, a set of dummy variables accounts for the level of adolescent autonomy and a domestic environment that facilitates learning (own room, desk to study, quiet place to study).

Besides the family realm, adolescent well-being is largely influenced by the interactions with peers and the school environment (Park and Huebner, 2005; Raboteg-Saric and Sakic, 2014; Marquez and Main, 2020). Therefore, this study includes controls for peer well-being, peer socio-economic background, and school atmosphere. Controlling for peer well-being can account for potential interpersonal spillover effects in well-being, as some studies claim that happiness might be contagious (Lundqvist and Dimberg, 1995; Tumen and Zeydanli, 2015). Moreover, controlling for peer-averages might help to mitigate the effect of unobservable factors that may otherwise bias cross-sectional estimates (Altonji and Mansfield, 2014). I further control for relative socio-economic status (SES) by including peer averages of home possessions and parental education.^7^ In adult studies it is usually found that SWB rises with own income and wealth, while it falls with peer income and wealth (Clark and Oswald, 1996; Clark et al., 2008).

In order to account for school atmosphere, this study includes four additional variables: a 4-item index of student competition, a 4-item index of student cooperation, and a 4-item index of teacher support. All indexes are provided by the OECD/PISA team, and are scaled so that a value of zero indicates the OECD average. A detailed description of each index item can be obtained from Table S1. Lastly, a student’s sense of belonging at school is measured using a 6-item index.

This study further controls for location effects with five dummies indicating the level of urbanization in which a student resides, and a full set of district dummies. Controlling for district fixed effects helps to control for within-country, local differences in culture and use of language that might influence the way well-being is experienced and expressed.

### 2.3 Summary statistics

Table 1 presents summary statistics for the sample of 67 countries and *N* = 376,641 15-year-old students for which information on all variables is available.^8^ Statistics have been adjusted using sampling weights, thus they represent population means and population standard deviations. Across the adolescent population, 51% are female, 25% are single children, and 6 % have a migrant background. At home, 80% of students have access to an own room, 84% have a desk to study, and 82% report to have a quiet place for study. Forty-three percent of students live in cities, 48% in towns, and 9 % in rural areas. Mean life satisfaction is 7.05 (SD 2.62). Happiness, positive affect, and negative affect have mean values of 3.39, 3.31, and 2.42 (SD 0.67, 0.58, and 0.60), respectively. Moreover, the average meaning in life index score is 0.19 (SD 0.97).

**Table 1:** Summary statistics.

Variable	Mean	Std. dev.	Min	Max
Subjective well-being				
Life satisfaction	7.05	2.61	0	10
Happiness	3.39	0.67	1	4
Positive affect	3.31	0.58	1	4
Negative affect	2.42	0.60	1	4
Meaning in life index	0.19	0.97	−2.15	1.74
Individual characteristics				
Female	0.51		0	1
Single child	0.25		0	1
Migrant background	0.06		0	1
Self-efficacy/resilience index	0.01	0.98	−3.17	2.77
Emotional interdependence	2.65	0.93	1	4
Family characteristics				
Parents max years of schooling	13.04	3.27	3	18
Home possessions index	−0.78	1.27	−9.49	5.92
Parents’ emotional support index	−0.02	1.00	−2.45	1.03
*Autonomy/learning environment at home*				
Own room	0.80		0	1
Desk to study	0.84		0	1
Quiet place to study	0.82		0	1
Peer characteristics				
Life satisfaction (peer avg.)	7.05	0.90	0	10
Happiness (peer avg.)	3.38	0.21	1	4
Positive affect (peer avg.)	3.30	0.20	1	4
Negative affect (peer avg.)	2.42	0.22	1	4
Meaning in life index (peer avg.)	0.18	0.35	−2.15	1.74
Home possessions index (peer avg.)	−0.80	0.95	−4.67	2.66
Parents max years of schooling (peer avg.)	13.02	1.96	3	18
School atmosphere				
Student competition index (school avg.)	0.12	0.36	−1.99	2.04
Student cooperation index (school avg.)	0.02	0.37	−2.14	1.68
Teacher support index (school avg.)	0.24	0.35	−2.15	1.31
Sense of belonging index	−0.11	0.93	−3.29	3.23
Level of urbanization				
Village	0.09		0	1
Small town	0.19		0	1
Town	0.29		0	1
City	0.27		0	1
Large city	0.16		0	1
World region				
Western Europe	0.06		0	1
Eastern Europe	0.03		0	1
Northern Europe	0.03		0	1
Southern Europe	0.05		0	1
United States	0.15		0	1
Confucian East Asia	0.14		0	1
Non-Confucian East Asia	0.26		0	1
Latin America	0.15		0	1
Middle East and North Africa	0.07		0	1
CIS	0.07		0	1

*N = 376,641. Population means calculated using official PISA 2018 survey weights.*

Given that multi-item indexes accounting for personality traits and relationships have been standardized by PISA with means of zero (= OECD average) and standard deviations of one, for illustration purposes it can be useful to discuss statistics of selected items included in these indexes. As one of the measures of self-efficacy/resilience, approximately four out of five adolescents agree (66.1%) or strongly agree (19.3%) to “usually manage one way or the other.” Emotional interdependence is measured by the survey item “When I am failing, I worry about what others think of me.” This statement is agreed to by 40.5% of adolescents, while 16.3% strongly agree. With regard to meaning in life, approximately three quarters of students agree (49.7%) or strongly agree (25.3%) to the statement “My life has clear meaning or purpose.” Furthermore, as one of the items in the “sense of belonging index,” 73.1% of students feel like they belong at school. Lastly, with regard to school atmosphere, 53.3 (62.8)% of students responded that it is “very true” or “extremely true” that students at their school are competing (cooperating) with each other.

### 2.4 Methodology

In order to analyze the predictors of well-being, $W_{isd}$, of student *i* who is attending school *s* in district *d*, the following empirical model will be employed:


(1) $$\begin{array}{l} W_{isd}=\alpha +\text{Famil}y_{isd}^{\prime }\beta +\text{GenderPersonalit}y_{isd}^{\prime }\gamma +\text{Relatio}n_{isd}^{\prime }\delta \\ \;+\text{Schoo}l_{sd}^{\prime }\zeta +\text{Urba}n_{sd}^{\prime }\theta +\mu_{d}+\varepsilon_{isd} \end{array}$$

where $\text{Famil}y_{isd}$ represents a vector of family background variables, $\text{GenderPersonalit}y_{isd}$ a vector of gender and personality traits, $\text{Relatio}n_{isd}$ a vector of relationship variables, $\text{Schoo}l_{sd}$ a vector of school atmosphere variables, and $\text{Urba}n_{sd}$ is a vector of location dummies accounting for the level of urbanization. Lastly, $\mu_{d}$ represents district (or in some models world region) fixed effects, and $\varepsilon_{isd}$ is the error term which is assumed to be well-behaved. Life satisfaction is measured on a 11-point scale from 0 to 10. Many studies have applied linear regression techniques to models similar to the one in Equation 1 (Ferrer-i-Carbonell and Frijters, 2004; Rudolf and Kang, 2015). I follow this strategy and estimate Equation 1 using OLS while correcting standard errors for clustering at the school level. Most variables in Equation 1 can be considered exogenous and thus determinants of well-being. However, one should be cautious with the interpretation of some of the RHS variables, in particular personality traits, relationship indexes, as well as peer well-being; these should be regarded as predictors rather than determinants since reverse causality cannot be ruled out.

Equation 1 can be estimated over the global PISA student sample in order to see how each set of factors predicts adolescent well-being globally. Moreover, two strategies will be employed to examine differences in the predictors of well-being across world regions. First, Equation 1 can be estimated separately for each world region, providing an indication of how the predictors of student well-being differ across groups of countries. Second, a Blinder-Oaxaca type decomposition will be used in order to account for life satisfaction differences between Confucian East Asia (CEA) and other world regions (Blinder, 1973; Senik, 2014).^9^

Students from CEA countries are of particular interest since they are usually found to excel in PISA, while at the same time ranking poorly with regards to subjective well-being. Differences in SWB between world regions can be due to (1) differences in endowments with SWB predictors such as individual characteristics, family characteristics, autonomy, peer characteristics, school atmosphere, etc.; (2) differences in how these predictors are related to SWB; and (3) the interactions between (1) and (2). Suppose we are interested in decomposing the mean life satisfaction difference *Δ* between CEA students and students from all other countries [for simplicity denoted as “rest of the world” (RW)] as in Equation (2)


(2) $$\Delta =E\left(LS_{CEA}\right)-E\left(LS_{RW}\right)$$

Following Jann (2008), substituting the linear model $E\left(LS\right)=E\left(X^{\prime }\beta +\varepsilon \right)$ into (2) and rearranging the equation, one obtains^10^


(3) $$\begin{array}{l} \Delta ={\left\{E\left(X_{CEA}\right)-E\left(X_{RW}\right)\right\}}^{\prime }\beta_{RW}+E{\left(X_{RW}\right)}^{\prime }\left(\beta_{CEA}-\beta_{RW}\right)\\ \;+{\left\{E\left(X_{CEA}\right)-E\left(X_{RW}\right)\right\}}^{\prime }\left(\beta_{CEA}-\beta_{RW}\right) \end{array}$$

Equation 3 is the Blinder-Oaxaca threefold decomposition that allows dividing the life satisfaction difference between two groups of countries into three components: contributions of differences in endowments (${\left\{E\left(X_{CEA}\right)-E\left(X_{RW}\right)\right\}}^{\prime }\beta_{RW}$), contributions of differences in coefficients ($E{\left(X_{RW}\right)}^{\prime }\left(\beta_{CEA}-\beta_{RW}\right)$), and contributions of interactions between endowments and coefficients (${\left\{E\left(X_{CEA}\right)-E\left(X_{RW}\right)\right\}}^{\prime }\left(\beta_{CEA}-\beta_{RW}\right)$).

## 3 Results

### 3.1 Predictors of well-being among 15-year-olds

Table 2 presents estimation results of Equation 1 across the global sample for life satisfaction, happiness, positive affect, negative affect, and meaning in life. Estimations were run over up to 398,609 students from up to 70 countries and explain between 18.7 and 27.6% of the variation in the five well-being measures.^11^

**Table 2:** Predictors of subjective well-being among adolescents.

	(1)	(2)	(3)	(4)	(5)
	Life satisfaction	Happiness	Positive affect	Negative affect	Meaning in life
Individual characteristics					
Female	−0.433***	0.00429**	−0.000269	0.250***	−0.0831***
	(0.00806)	(0.00214)	(0.00182)	(0.00198)	(0.00289)
Single child	−0.105***	−0.0198***	−0.0342***	0.0135***	−0.0843***
	(0.00909)	(0.00241)	(0.00202)	(0.00210)	(0.00337)
Migrant background	−0.136***	−0.0131***	−0.0116***	−0.000641	0.0230***
	(0.0160)	(0.00418)	(0.00344)	(0.00369)	(0.00551)
Self-efficacy/resilience index	0.395***	0.120***	0.130***	−0.0527***	0.333***
	(0.00498)	(0.00134)	(0.00114)	(0.00123)	(0.00179)
Emotional interdependence	−0.207***	−0.0221***	−0.0188***	0.122***	−0.0111***
	(0.00462)	(0.00123)	(0.00103)	(0.00111)	(0.00172)
Family characteristics					
Parents max years of schooling	−0.00988***	−0.00416***	−0.00350***	0.00109***	−0.00391***
	(0.00158)	(0.000415)	(0.000343)	(0.000359)	(0.000529)
Home possessions index	0.849***	0.00906***	0.0106***	0.0132***	0.00551***
	(0.00558)	(0.00150)	(0.00127)	(0.00131)	(0.00188)
Parents’ emotional support index	0.383***	0.0890***	0.0755***	−0.0254***	0.139***
	(0.00477)	(0.00123)	(0.00104)	(0.00103)	(0.00167)
Autonomy/learning environment at home					
Own room	0.131***	0.0221***	0.0150***	−0.0203***	0.0102***
	(0.0112)	(0.00292)	(0.00240)	(0.00247)	(0.00385)
Desk to study	0.133***	0.0386***	0.0350***	−0.0105***	0.0234***
	(0.0160)	(0.00409)	(0.00331)	(0.00345)	(0.00502)
Quiet place to study	0.642***	0.119***	0.0964***	−0.0628***	0.0858***
	(0.0147)	(0.00380)	(0.00309)	(0.00314)	(0.00454)
Peer characteristics					
Outcome variable (peer avg.)	0.219***	0.142***	0.178***	0.150***	0.143***
	(0.00861)	(0.00980)	(0.00871)	(0.00850)	(0.00819)
Home possessions index (peer avg.)	−0.222***	−0.0109***	−0.0288***	0.0175***	−0.0755***
	(0.0146)	(0.00401)	(0.00325)	(0.00346)	(0.00510)
Parents max years of schooling (peer avg.)	−0.0202***	−0.00416***	−0.00365***	0.00624***	−0.0124***
	(0.00506)	(0.00139)	(0.00114)	(0.00119)	(0.00180)
School atmosphere					
Student competition index	−0.0380**	−0.00831*	−0.00800**	0.0228***	0.0132**
(school avg.)	(0.0160)	(0.00437)	(0.00357)	(0.00387)	(0.00586)
Student cooperation index	0.0687***	0.0256***	0.0241***	−0.00321	−0.000958
(school avg.)	(0.0153)	(0.00419)	(0.00350)	(0.00348)	(0.00555)
Teacher support index	0.112***	0.00979***	0.00427	−0.0146***	0.0377***
(school avg.)	(0.0139)	(0.00375)	(0.00313)	(0.00321)	(0.00512)
Sense of belonging index	0.436***	0.130***	0.131***	−0.108***	0.117***
	(0.00461)	(0.00119)	(0.00106)	(0.00115)	(0.00176)
Level of urbanization					
Village	Reference	Reference	Reference	Reference	Reference
Small town	−0.0592***	−0.00255	−0.00365	0.00693*	−0.00466
	(0.0173)	(0.00471)	(0.00385)	(0.00400)	(0.00576)
Town	−0.0769***	−0.00458	−0.0103***	0.00895**	−0.00987*
	(0.0171)	(0.00467)	(0.00386)	(0.00397)	(0.00579)
City	−0.138***	−0.0153***	−0.0207***	0.0181***	−0.0294***
	(0.0176)	(0.00485)	(0.00398)	(0.00414)	(0.00601)
Large city	−0.109***	−0.00768	−0.0219***	0.00965**	−0.0323***
	(0.0205)	(0.00563)	(0.00464)	(0.00481)	(0.00703)
District FE	Yes	Yes	Yes	Yes	Yes
Constant	5.832***	2.938***	2.680***	1.567***	0.268***
	(0.119)	(0.0421)	(0.0364)	(0.0320)	(0.0408)
Observations	376,641	387,079	388,054	387,195	398,609
No of countries	67	68	68	68	70
R-squared	0.217	0.187	0.246	0.239	0.276

OLS estimation. Robust standard errors in parentheses (corrected for clustering at the school level); ***p < 0.01, **p < 0.05, *p < 0.1.

#### 3.1.1 Individual characteristics

Results indicate that being female significantly reduces life satisfaction and meaning in life for girls, while it increases affect. This is in line with earlier studies (e.g., Bergman and Scott, 2001; Lee and Yoo, 2015; Rudolf and Bethmann, 2023). In particular, being a girl reduces life satisfaction by 0.433 (*p* = 0.000) or 16.6% of a standard deviation, while it raises negative affect by 0.250 (*p* = 0.000) or 41.7% of a standard deviation. Positive affect does not differ by gender, except for the feeling of happiness which is slightly more reported by girls. Being a girl further reduces perceived meaning in life by −0.0831 (*p* = 0.000). Moreover, being a single child and having migration background both reduce adolescent well-being, although effects are not particularly large. With regard to personality traits, higher levels of self-efficacy and lower levels of emotional interdependence are found to be associated with higher well-being among sample participants. Both variables’ effects are large in size and highly statistically significant [e.g., +0.395 (*p* = 0.000) and − 0.207 (*p* = 0.000) association with life satisfaction].

#### 3.1.2 Family characteristics

Parental education is found to exert small, but statistically significant negative effects on adolescent well-being, holding everything else constant. On the contrary, the level of home possessions and parents’ emotional support are both positively related to the well-being of adolescents. The latter in particular shows a large and highly significant effect indicating the key importance of the child–parent relationship.

#### 3.1.3 Autonomy/learning environment at home

Having an own room, a desk to study, and a quiet place to study at home all show significant positive impacts on student well-being. All effects are statistically significant at the 1-percent significance level. Remarkable are the large coefficient magnitudes of having a quiet place to study; e.g. it increases life satisfaction by 0.642 (*p* = 0.000) or 24.6% of a standard deviation.

#### 3.1.4 Peer characteristics

Peer effects play important roles for individual well-being. Peer averages of the outcome variables are all statistically significant at the 1-percent level. Given that all models control for district dummies which should capture cultural and language differences across space, significant peer well-being effects suggest spillovers in well-being across school peers. Besides peer well-being, adolescent’s well-being is influenced by peers via relative SES. Table 2 results confirm that higher education and wealth of peer families reduce individual well-being among adolescents. This expresses itself in lower life satisfaction, less frequent experiences of positive emotions, more frequent experiences of negative emotions, as well as lower levels of meaning and purpose perceived in life. The finding that the (negative) effect of peer home possessions is larger than the (positive) effect of own home possessions suggests that relative wealth is more important than absolute wealth also for adolescents, a common finding in adult SWB studies (e.g., Clark et al., 2008). Moreover, that SWB is reduced in schools with students from wealthier and more educated backgrounds is in line with Rudolf and Lee (2023) who show that when the average education level of parents is high, within-school competition increases which adversely affects adolescent life satisfaction.

#### 3.1.5 School atmosphere

Although featuring rather small coefficient magnitudes, competition among students is found to reduce well-being, while cooperation increases it. Students are further found to benefit from higher levels of teacher support. In addition, large effects are found for the association between sense of belonging at school and students’ well-being which is in line with Chiu et al. (2016).

#### 3.1.6 Location

Regarding urbanization effects, *ceteris paribus*, a higher level of urbanization is associated with lower levels of well-being, although effect sizes are rather moderate. Lastly, district fixed effects included were found significant in many cases indicating important differences in well-being and its expression across space.

### 3.2 Life satisfaction among 15-year-olds by world region

#### 3.2.1 Regional differences in well-being and in the endowment with its predictors

Table 3 presents mean values of the variables used in the prior analysis, yet now disaggregated by 10 major world regions.^12^ Mean life satisfaction among adolescents is found to be highest in CIS^13^ countries (7.52 [SD 2.62]), Latin America (7.45 [SD 2.64]) and Non-Confucian East Asia (7.40 [SD 2.50]), while it is lowest in the Middle East and North Africa (MENA; 6.29 [SD 3.11]), Northern Europe (6.42 [SD 2.63]), and Confucian East Asia (6.44 [SD 2.55]). While not presented in the table, most of these regional differences are highly statistically significant.

**Table 3:** SWB and endowments with its predictors by world region.

	(1)	(2)	(3)	(4)	(5)	(6)	(7)	(8)	(9)	(10)
	Western Europe	Eastern Europe	Northern Europe	Southern Europe	United States	Confucian East Asia	Non-Confucian East Asia	Latin America	Middle East and North Africa	CIS
Subjective well-being										
Life satisfaction	7.20	7.11	6.42	7.21	6.76	6.44	7.40	7.45	6.29	7.52
Happiness	3.40	3.28	3.28	3.38	3.35	3.40	3.49	3.43	3.17	3.30
Positive affect	3.31	3.27	3.14	3.24	3.18	3.30	3.47	3.37	3.11	3.24
Negative affect	2.35	2.33	2.51	2.36	2.40	2.66	2.53	2.33	2.23	2.13
Meaning in life index	0.07	−0.01	−0.20	0.04	0.11	−0.13	0.48	0.28	0.25	0.17
Individual characteristics										
Female	0.50	0.51	0.53	0.50	0.50	0.50	0.52	0.52	0.51	0.52
Single child	0.33	0.31	0.29	0.36	0.24	0.32	0.06	0.40	0.38	0.25
Migrant background	0.15	0.01	0.15	0.09	0.21	0.01	0.01	0.01	0.06	0.06
Self-efficacy/resilience index	−0.05	0.00	−0.12	0.11	0.16	−0.30	0.00	0.16	0.33	−0.24
Emotional interdependence	2.40	2.52	2.73	2.51	2.66	2.97	2.72	2.49	2.61	2.48
Family characteristics										
Parents max years of schooling	14.13	13.57	14.20	13.74	14.16	13.36	11.73	12.79	11.63	14.63
Home possessions index	0.09	−0.18	0.32	−0.09	0.07	−0.42	−1.79	−1.19	−1.08	−0.43
Parents’ emotional support index	0.09	−0.20	0.06	−0.02	0.09	−0.10	0.01	−0.02	−0.01	−0.30
Autonomy/learning environment at home										
Own room	0.92	0.87	0.90	0.79	0.88	0.88	0.75	0.68	0.72	0.83
Desk to study	0.97	0.96	0.91	0.96	0.80	0.95	0.77	0.74	0.77	0.97
Quiet place to study	0.95	0.94	0.91	0.92	0.90	0.89	0.66	0.79	0.86	0.90
Peer characteristics										
Life satisfaction (peer avg.)	7.20	7.11	6.45	7.20	6.75	6.43	7.38	7.46	6.32	7.53
Happiness (peer avg.)	3.39	3.27	3.27	3.38	3.35	3.40	3.48	3.43	3.16	3.29
Positive affect (peer avg.)	3.31	3.26	3.14	3.23	3.18	3.30	3.45	3.37	3.10	3.23
Negative affect (peer avg.)	2.34	2.33	2.49	2.37	2.40	2.65	2.53	2.32	2.24	2.13
Meaning in life index (peer avg.)	0.07	−0.02	−0.20	0.03	0.11	−0.13	0.47	0.27	0.24	0.16
Home possessions index (peer avg.)	0.07	−0.19	0.29	−0.10	0.05	−0.42	−1.80	−1.23	−1.10	−0.43
Parents max years of schooling (peer avg.)	14.12	13.56	14.20	13.74	14.16	13.36	11.75	12.67	11.59	14.60
School atmosphere										
Student competition index (school avg.)	−0.26	0.03	0.25	−0.07	0.39	0.05	0.17	0.03	0.32	−0.10
Student cooperation index (school avg.)	−0.12	−0.04	−0.10	−0.14	−0.16	0.16	0.30	−0.22	−0.04	0.01
Teacher support index (school avg.)	−0.29	−0.10	0.27	0.07	0.14	0.19	0.40	0.40	0.30	0.23
Sense of belonging index	0.13	−0.15	−0.16	0.20	−0.23	−0.02	−0.16	−0.08	−0.10	−0.33
Level of urbanization										
Village	0.02	0.18	0.08	0.03	0.06	0.02	0.18	0.06	0.04	0.16
Small town	0.25	0.18	0.23	0.20	0.18	0.10	0.30	0.13	0.08	0.14
Town	0.47	0.35	0.35	0.45	0.36	0.21	0.26	0.27	0.22	0.21
City	0.19	0.22	0.25	0.23	0.27	0.38	0.20	0.32	0.28	0.31
Large city	0.06	0.08	0.10	0.09	0.13	0.29	0.06	0.22	0.38	0.17
Observations	23,299	31,856	36,050	82,422	4,018	38,525	33,763	41,050	45,988	39,670

Population means calculated using official PISA 2018 survey weights.

Table 3 results suggest that students in MENA countries show on average the lowest levels of life satisfaction, happiness, and positive affect across all country groups. Interestingly, they also report the second lowest level of negative emotions. Average happiness and positive affect are highest among students in non-Confucian East Asia (NCEA), followed by Latin America. Interestingly, CEA, which shows low levels of life satisfaction and the highest level of negative affect, is in the upper half of world regions when it comes to the feeling of happiness and positive affect. Average reported happiness is lowest in Northern Europe and the MENA region. Lastly, perceived meaning in life is lower in high-income countries (columns 1–6) compared to middle-income countries (columns 7–10).

The endowment with the factors associated with well-being can also differ markedly across world regions, partly explaining differences in SWB. Socio-economic characteristics such as home possessions and facilities for study at home are higher in high-income regions compared to middle-income regions. These factors are known to be positively correlated with SWB. However, high-income regions also face a higher share of students with migrant background and higher levels of parental education, both of which tend to decrease personal well-being according to Table 2.

With regards to personality factors, self-efficacy is found to be highest among students in the MENA region, as well as in the US and in Latin America. Emotional interdependence is lowest among Western European students, while it is found to be highest among Confucian East Asians, confirming earlier literature (Suh and Choi, 2018). Being fully aware of the potential of reverse causality, earlier literature has suggested that the endowment with personality-related factors, that are likely to be a product of East Asian cultural norms and values, puts CEA students at a disadvantage when it comes to the generation of well-being (Stankov, 2010; Suh and Choi, 2018). Table 3 shows that indeed, students from this region show *lowest* average levels of self-efficacy (−0.300), and *highest* average levels of emotional interdependence (2.971) across all 10 world regions.

Turning to relationship factors, CIS countries show lowest parental emotional support (−0.301) and sense of belonging at school (−0.327). Parents’ emotional support is also perceived relatively low in Eastern Europe and CEA, while sense of belonging at school is surprisingly low in the US (−0.233). With regard to school atmosphere, competition levels among students are perceived to be highest among adolescents in the US (0.387) and Northern Europe (0.253), and lowest among students in Western Europe (−0.261). Cooperation is perceived to be highest in East Asia, and lowest in Latin America. Finally, levels of teacher support are perceived to be higher in middle-income regions compared to high-income regions.

#### 3.2.2 Region-specific associations between predictors and adolescent well-being

As found in earlier literature, the importance that people place on various aspects of their lives when making life satisfaction judgments differs across cultures (Chen et al., 2015; Suh and Choi, 2018). Table 4 presents the results of estimating Equation 1 by world region. Regression models explain between 15.3% (NCEA) and 29.0% (Northern Europe) of the variation in adolescent life satisfaction.^14^ Regional models largely confirm earlier findings from Table 2, yet reveal interesting region-specific differences. Several observations can be made in particular:

- Gender and migrant background: Across all world regions, girls show lower life satisfaction compared to boys at the age of 15. Interestingly, the gender gap in adolescent life satisfaction is largest in Europe [e.g., −0.574 (*p* = 0.000) for Western Europe], while it is lowest in East Asia [e.g., −0.166 (*p* = 0.000) for NCEA; −0.240 (*p* = 0.000) for CEA]. These findings stand in opposition to global rankings of gender equality. Yet, they may imply that girls in East Asia experience less school-related stress in teen age given that they are not expected to prepare for the breadwinner role in later life. Moreover, results indicate that being a single child has a stronger negative effect on adolescent life satisfaction in countries with higher fertility rates. In contrast, in Europe and Confucian East Asia, where fertility rates are among the lowest in the world, being a single child seems to be more socially accepted. Having a migration history in the family reduces life satisfaction most strongly in Eastern and Southern Europe, as well as in MENA countries.
- *Personality traits*: Both traits are highly significant across all regions. Among all regions, CEA adolescents’ life satisfaction appears to be most responsive to emotional interdependence [−0.337 (*p* = 0.000)]. Moreover, self-efficacy is most strongly linked to life satisfaction for European adolescents.
- *Family characteristics*: Home possessions have positive and statistically significant effects in all regions except for CEA. Moreover, region-specific estimations reveal that parental education is significantly and negatively linked to adolescent life satisfaction only for middle-income regions, yet not for high-income regions. Being part of the education elite in middle-income countries might imply a stronger pressure on adolescents compared to students in high-income countries, since more is at stake for the former.
- *Autonomy/learning environment at home*: Both having an own room and a desk to study at home are positively and significantly linked to life satisfaction in the majority of world regions. CEA and the United States are the only two regions in which these two variables are statistically insignificant. The most salient factor, however, is having access to a quiet place to study a home. The latter increases life satisfaction significantly across all regions, has large effects, and can be linked to earlier literature on autonomy and self-determination (Chen et al., 2015; Ryan and Deci, 2017).
- *Relationships*: Parents’ emotional support and the sense of belonging at school are both strongly associated with life satisfaction across all world regions. These findings stand in opposition to earlier literature that suggested emotional support to be more relevant to East Asian students compared to European Americans (e.g., Uchida et al., 2008). Instead, I find that both US and CEA students show the highest links between relational factors (parents’ emotional support; sense of belonging at school) and life satisfaction across all world regions. This suggests that a too simple individualistic/collectivistic classification is not able to capture the complexities of cultural differences.
- *Peer characteristics*: School peers’ average life satisfaction positively affects individual life satisfaction across all world regions, *ceteris paribus*. With regard to relative SES, peer wealth and peer parental education affect adolescent well-being negatively and statistically significantly in many world regions. Again, CEA stands out; it is the only region in which peer wealth does not affect individual well-being significantly.
- *School atmosphere*: Student competition has a statistically significant negative effect only for students in CEA, but in no other world region. Student cooperation also matters most for CEA students, confirming earlier literature on East Asian “collectivistic” cultures.
- *Urbanization*: Except for MENA and East Asia (both CEA and NCEA), the degree of urbanization shows a negative relationship with life satisfaction. The negative association between living in a city and life satisfaction is strongest for adolescents residing in the United States.

**Table 4:** Predictors of subjective well-being among adolescents—estimations by world region.

Outcome variable: life satisfaction										
	(1)	(2)	(3)	(4)	(5)	(6)	(7)	(8)	(9)	(10)
	Western Europe	Eastern Europe	Northern Europe	Southern Europe	United States	Confucian East Asia	Non-Confucian East Asia	Latin America	Middle East and North Africa	CIS
Individual characteristics										
Female	−0.574***	−0.461***	−0.504***	−0.555***	−0.429***	−0.240***	−0.166***	−0.456***	−0.406***	−0.362***
	(0.0290)	(0.0280)	(0.0235)	(0.0165)	(0.0762)	(0.0243)	(0.0262)	(0.0232)	(0.0281)	(0.0238)
Single child	−0.0449	−0.0606**	−0.0742***	−0.106***	−0.166**	−0.0835***	−0.149***	−0.143***	−0.115***	−0.143***
	(0.0292)	(0.0285)	(0.0282)	(0.0177)	(0.0740)	(0.0276)	(0.0498)	(0.0253)	(0.0348)	(0.0288)
Migrant background	−0.0963**	−0.252*	−0.0682	−0.212***	−0.111	−0.0992**	−0.122	−0.0216	−0.154***	−0.0127
	(0.0403)	(0.129)	(0.0454)	(0.0329)	(0.101)	(0.0459)	(0.0992)	(0.0642)	(0.0375)	(0.0528)
Self-efficacy/resilience	0.505***	0.479***	0.580***	0.418***	0.375***	0.373***	0.313***	0.431***	0.239***	0.284***
index	(0.0169)	(0.0180)	(0.0150)	(0.0105)	(0.0421)	(0.0157)	(0.0175)	(0.0131)	(0.0149)	(0.0143)
Emotional interdependence	−0.228***	−0.263***	−0.284***	−0.194***	−0.265***	−0.337***	−0.154***	−0.152***	−0.151***	−0.115***
	(0.0147)	(0.0162)	(0.0140)	(0.00943)	(0.0353)	(0.0165)	(0.0166)	(0.0125)	(0.0142)	(0.0150)
Family characteristics										
Parents max years of	−0.0111*	0.00139	−0.00334	−0.00262	−0.00605	−0.00545	−0.0126**	−0.0170***	−0.0174***	−0.0240***
schooling	(0.00605)	(0.00587)	(0.00585)	(0.00286)	(0.0161)	(0.00511)	(0.00500)	(0.00389)	(0.00503)	(0.00858)
Home possessions index	0.0585***	0.0834***	0.0652***	0.0723***	0.0920**	0.0136	0.0833***	0.0666***	0.168***	0.110***
	(0.0214)	(0.0207)	(0.0174)	(0.0120)	(0.0428)	(0.0171)	(0.0165)	(0.0162)	(0.0170)	(0.0176)
Parents’ emotional support index	0.386***	0.363***	0.391***	0.382***	0.510***	0.449***	0.292***	0.428***	0.420***	0.284***
	(0.0173)	(0.0168)	(0.0138)	(0.00993)	(0.0436)	(0.0148)	(0.0166)	(0.0128)	(0.0158)	(0.0135)
*Autonomy/learning environment at home*										
Own room	0.00269	0.151***	0.0158	0.158***	−0.0338	−0.0295	0.124***	0.0962***	0.146***	0.283***
	(0.0582)	(0.0393)	(0.0410)	(0.0252)	(0.120)	(0.0330)	(0.0311)	(0.0278)	(0.0328)	(0.0339)
Desk to study	0.211**	0.212**	0.260***	0.189***	−0.0488	0.0603	0.163***	0.0611*	0.128***	0.0272
	(0.0854)	(0.0844)	(0.0561)	(0.0481)	(0.0972)	(0.0545)	(0.0383)	(0.0321)	(0.0386)	(0.0782)
Quiet place to study	0.868***	0.707***	0.828***	0.768***	0.758***	0.601***	0.206***	0.696***	0.798***	0.835***
	(0.0808)	(0.0586)	(0.0535)	(0.0377)	(0.137)	(0.0394)	(0.0299)	(0.0354)	(0.0432)	(0.0517)
Peer characteristics										
Life satisfaction (peer avg.)	0.158***	0.150***	0.0920***	0.215***	0.178**	0.171***	0.349***	0.119***	0.301***	0.223***
	(0.0335)	(0.0266)	(0.0262)	(0.0183)	(0.0769)	(0.0300)	(0.0298)	(0.0246)	(0.0247)	(0.0264)
Home possessions index	−0.111	−0.127**	−0.0123	−0.238***	−0.267**	−0.0799	−0.280***	−0.246***	−0.220***	−0.309***
(peer avg.)	(0.0728)	(0.0558)	(0.0538)	(0.0359)	(0.115)	(0.0497)	(0.0350)	(0.0377)	(0.0380)	(0.0471)
Parents max years of schooling	−0.0112	−0.0431**	−0.0106	−0.0100	0.0508	−0.0417***	−0.00906	−0.0154	−0.0324**	−0.0560**
(peer avg.)	(0.0237)	(0.0193)	(0.0224)	(0.00982)	(0.0564)	(0.0143)	(0.0144)	(0.0134)	(0.0152)	(0.0275)
School atmosphere										
Student competition index	−0.0379	−0.0744	−0.0169	−0.0108	−1.17e-05	−0.285***	−0.0337	−0.00517	0.0453	−0.0363
(school avg.)	(0.0598)	(0.0583)	(0.0496)	(0.0325)	(0.172)	(0.0482)	(0.0597)	(0.0450)	(0.0590)	(0.0477)
Student cooperation index	−0.0173	0.121**	0.130***	0.0159	0.179	0.172***	0.0418	0.118***	0.0385	0.0343
(school avg.)	(0.0589)	(0.0486)	(0.0482)	(0.0308)	(0.167)	(0.0611)	(0.0596)	(0.0386)	(0.0569)	(0.0475)
Teacher support index	0.105**	0.179***	0.0389	0.0515*	0.224	0.0557	0.0463	0.0695*	0.202***	0.226***
(school avg.)	(0.0494)	(0.0404)	(0.0404)	(0.0269)	(0.149)	(0.0588)	(0.0516)	(0.0397)	(0.0499)	(0.0519)
Sense of belonging index	0.413***	0.465***	0.505***	0.364***	0.628***	0.641***	0.494***	0.391***	0.377***	0.388***
	(0.0157)	(0.0161)	(0.0141)	(0.00872)	(0.0422)	(0.0158)	(0.0188)	(0.0120)	(0.0156)	(0.0145)
Level of urbanization										
Village	Reference	Reference	Reference	Reference	Reference	Reference	Reference	Reference	Reference	Reference
Small town	−0.162*	0.00234	−0.116**	−0.140***	−0.112	−0.118	0.00538	0.0706	0.0146	−0.0871*
	(0.0887)	(0.0535)	(0.0457)	(0.0428)	(0.136)	(0.0879)	(0.0400)	(0.0536)	(0.0557)	(0.0464)
Town	−0.168*	−0.0136	−0.137***	−0.163***	−0.182	0.0141	0.00420	0.0279	−0.0408	−0.126***
	(0.0891)	(0.0534)	(0.0462)	(0.0431)	(0.123)	(0.0812)	(0.0407)	(0.0526)	(0.0566)	(0.0450)
City	−0.183**	−0.0717	−0.176***	−0.217***	−0.307**	−0.0956	−0.0232	−0.0750	−0.0622	−0.205***
	(0.0906)	(0.0598)	(0.0497)	(0.0449)	(0.138)	(0.0815)	(0.0421)	(0.0549)	(0.0553)	(0.0419)
Large city	−0.164	−0.137*	−0.297***	−0.203***	−0.284**	−0.0475	0.0371	−0.122**	−0.000388	−0.143***
	(0.108)	(0.0780)	(0.0900)	(0.0558)	(0.143)	(0.0828)	(0.0515)	(0.0577)	(0.0571)	(0.0546)
District FE	YES	YES	YES	YES	YES	YES	YES	YES	YES	YES
Constant	5.942***	7.323***	7.131***	6.007***	5.580***	7.032***	5.289***	8.289***	3.791***	6.574***
	(0.482)	(0.396)	(0.389)	(0.351)	(1.035)	(0.357)	(0.305)	(0.301)	(0.257)	(0.505)
Observations	23,299	31,856	36,050	82,422	4,018	38,525	33,763	41,050	45,988	39,670
R-squared	0.227	0.195	0.290	0.209	0.263	0.217	0.153	0.208	0.172	0.194

OLS estimation. Robust standard errors in parentheses (corrected for clustering on the school level); ***p < 0.01, **p < 0.05, *p < 0.1.

### 3.3 Decomposing the adolescent life satisfaction gap between Confucian East Asia and all other regions

Blinder-Oaxaca decomposition techniques can be employed to shed additional light on mean life satisfaction differences between world regions. I use a threefold decomposition adjusting for sampling weights (Jann, 2008). The region of particular interest, Confucian East Asia (CEA), tends to rank high in academic performance, yet low in SWB (Helliwell et al., 2018; OECD, 2019). Table 5 shows the results of a Blinder-Oaxaca decomposition of mean differences in life satisfaction (cp. Equation 3).

**Table 5:** Blinder-Oaxaca decomposition of the adolescent life satisfaction gap between Confucian East Asia and the rest of the world.

	LS (Rest of world) - LS (Conf. East Asia)		
Total gap	= 7.154–6.436 = 0.717***		
Explained by:			
Endowments	0.664***		
Coefficients	0.323***		
Interactions	−0.269**		
	Endowments	Coefficients	Interaction
	(1)	(2)	(3)
Individual characteristics			
Female	−0.00313*	−0.0818***	−0.00266
Single child	0.00143	−0.0322*	0.00744*
Migrant background	−0.00799	−0.000159	−0.00142
Self-efficacy/resilience index	0.135***	−0.00505	0.00610
Emotional interdependence	0.113***	0.347***	−0.0434***
Family characteristics			
Parents max years of schooling	0.00254	−0.0594	0.00166
Home possessions index	0.00308	−0.0439***	−0.0444***
Parents’ emotional support index	0.0357***	0.00312	−0.00268
Autonomy/learning environment at home			
Own room	0.00887*	0.158***	−0.0171***
Desk to study	0.00231	0.0791	−0.0110
Quiet place to study	−0.0523***	−0.172***	0.0150***
Peer characteristics			
Life satisfaction (peer avg.)	0.172***	0.572*	0.0644*
Home possessions index (peer avg.)	0.0850***	0.0122	0.0128
Parents max years of schooling (peer avg.)	0.00961	0.262	−0.00776
School atmosphere			
Student competition index (school avg.)	−0.0252***	0.00956*	0.0183**
Student cooperation index (school avg.)	−0.0170	−0.00707	0.00724
Teacher support index (school avg.)	0.00553	−0.00786	−0.00235
Sense of belonging index	−0.0751***	0.00561**	0.0256***
Level of urbanization			
Small town	−0.00147	−0.0111	−0.0108
Town	0.0109	−0.0601**	−0.0260*
City	−0.00493	−0.0932*	0.0314*
Large city	−0.00991	−0.0922**	0.0475**
Sum of country FE	0.276***	0.276***	−0.337***
Constant		−0.737	
Total	0.664***	0.323***	−0.269**

*N* = 376,641. Blinder-Oaxaca threefold decomposition of mean life satisfaction differences (population means using official PISA 2018 survey weights). Standard errors are corrected for clustering at the school level. ***p < 0.01, **p < 0.05, *p < 0.1. Estimations use country FE since district FE did not converge.

The total life satisfaction difference between students from all other countries (hereafter: rest of the world) and CEA is 0.717 (*p* = 0.000). Most of this difference can be explained by differences in endowments [0.664 (*p* = 0.000)] and coefficients [0.323 (*p* = 0.000)], while interactions of endowments and coefficients work in favor of students from CEA countries [−0.269 (*p* = 0.023)]. Two major groups of predictors drive the endowment effect. First, endowments in personality (lower self-efficacy, higher emotional interdependence in CEA) collectively account for 34.6% of the entire life satisfaction gap. That is, if CEA countries had the same levels of self-efficacy and emotional interdependence as all other countries, their predicted life satisfaction would rise significantly. Second, endowments in environmental factors such as peer life satisfaction, peer home possessions, and country effects jointly account for 74.3% of the life satisfaction gap. The latter figure suggests that besides socio-economic factors, cultural factors play a large role in shaping the playing field of adolescent well-being.

With regard to coefficient differences, emotional interdependence plays another important role. As seen in Table 4, worrying about what other’s think in case of one’s failure (emotional interdependence) only reduces life satisfaction in CEA countries, yet not in any other world region. Decomposition results suggest that in the absence of this unique effect, CEA adolescents’ life satisfaction would increase by 0.347, or 48.4% of the entire life satisfaction gap between CEA and the rest of the world. Another variable that matters for coefficient effects is urbanization. Living in urban areas is not associated with negative well-being effects for CEA adolescents, yet it is in most other world regions. This effect tends to reduce the life satisfaction gap between CEA and the rest of the world.

## 4 Discussion and conclusion

This study used 2018 PISA data from 70 countries across 10 world regions. The estimation sample employed is one of the largest used in the literature to date. New evidence to at least three rapidly growing threads of literature is provided: adolescent well-being, the cultural making of subjective well-being, and self-determination theory. This study’s findings on the global predictors of adolescent well-being largely confirm the findings of earlier research, in particular the important roles played by gender, personality and relationships (Levin and Currie, 2010; Morgan et al., 2012; Lee and Yoo, 2015; Coupe and Obrizan, 2018; Rudolf and Lee, 2023). Being a girl reduces life satisfaction significantly at age 15 (−0.166 SD), while being emotionally independent and having high self-efficacy are associated with significantly higher levels of adolescent SWB. With regards to relationship quality, parents’ emotional support and sense of belonging at school show strong and highly significant links with adolescent SWB. Results further indicate that socio-economic factors matter primarily *relative* to peers, confirming findings from adult SWB research that relative income matters more for individual well-being than absolute income (Clark et al., 2008). This study finds surprisingly strong effects of the predictors related to adolescent autonomy and the home learning environment, contributing new evidence in favor of self-determination theory (Ryan and Deci, 2000, 2017; Chen et al., 2015). In particular, having access to a quiet place to study at home is associated with a particularly high increase in life satisfaction (+0.246 SD). Having an own room and a desk to study at home are also significant predictors of adolescent SWB. This study further finds that peer well-being matters even after controlling for district fixed effects, suggesting spillovers in adolescent well-being. Lastly, student well-being is negatively related to living in cities.

The present study extends the literature on cultural differences in the factors that determine life satisfaction to 10 major world regions, focusing on the numerous particularities of Confucian East Asian countries. Low levels of life satisfaction among students from CEA are found to be associated with low self-efficacy, low peer well-being, as well as with high emotional interdependence compared to other world regions. CEA students exhibit the highest average levels of worry about peer reactions to individual failure. Moreover, these worries are more strongly linked to life satisfaction in CEA than in any other world region. In addition, findings indicate that competition and cooperation among students matters most strongly for adolescent life satisfaction in CEA countries. Findings further indicate that both US and CEA students show stronger links between relational factors (parents’ emotional support; sense of belonging at school) and life satisfaction compared to all other world regions. These findings mostly confirm earlier research suggesting that members of Confucian East Asian /collectivist cultures place more importance on relational factors, particularly family and friends (Diener and Diener, 1995; Suh et al., 1998; Park and Huebner, 2005). Especially my findings on emotional interdependence are a strong empirical test for Suh and Choi’s (2018) hypothesis that “East Asians’ excessive concern of other’s view might be a key psychological reason for why their happiness level is lower than expected by economic indices.” This study’s results further confirm Stankov (2010) who argued that high academic achievement of students from Confucian Asian countries is accompanied by high levels of anxiety and self-doubt. According to him, an “unforgiving” Confucian culture, coupled with the belief that effort rather than ability is the primary source of success, might be causing this phenomenon.

This paper’s findings, however, stand in opposition to earlier literature that suggested emotional support to be more relevant to East Asian students compared to European Americans in the generation of student well-being (Uchida et al., 2008). Instead, I find that relational factors play similarly important roles in both CEA and the US. Results further indicate that CEA is the only region in which neither own nor peer wealth affect adolescent life satisfaction. This suggests that a too simple individualistic/collectivistic classification is not able to capture the real complexities of cultural differences.

While the author is confident that this study adds significant new evidence to the existing literature, it does not come without limitations. Firstly, personality traits covered in PISA are not all-encompassing. This could lead to an overestimation of the effects of the two traits included in the model. To the extent that data allows, future studies should therefore control for a more complete list of personality traits. Secondly, estimated effects for some variables present correlations rather than causal effects. This is particularly true for personality and relationship variables. Thus, these effects should strictly be interpreted as predictors rather than determinants of well-being. Future research could from improvements in the quality of data by (1) collecting internationally harmonized longitudinal survey data, and (2) by conducting internationally harmonized in-school student experiments.

This study’s findings suggest that parents, educators and policymakers around the world should focus on creating a positive school environment that promotes well-being, student self-efficacy, a sense of belonging, and a safe space in which failure is accepted as part of the learning process. Such efforts are particularly needed in Confucian East Asian countries where students often face very high performance expectations. Moreover, extra support should be given to girls given their relatively lower SWB levels compared to boys.

## Data Availability

Publicly available datasets were analyzed in this study. This data can be found at: https://www.oecd.org/pisa/data/2018database/.

## Footnotes

^1^This study uses the term “Confucian East Asia” when referring to countries that have been largely influenced by Confucianism throughout their history. This includes China and its offshoots (Singapore, Hong Kong, Taiwan/Taipei, Macao), as well as Korea, Japan, and Vietnam. Non-Confucian East Asia are countries located in Southeast Asia other than Vietnam and Singapore.

^2^PISA 2018 data is preferred over PISA 2022 data, since affective well-being and meaning in life were not measured in PISA 2022.

^3^Due to space limitations, this paper’s analysis will focus on the cognitive aspect of SWB, i.e., life satisfaction.

^4^According to PISA’s survey design, 150 schools were surveyed in each country. Countries taking the computer-based assessment were supposed to sample 42 students from each of the 150 sampled schools, while countries taking the paper-based assessment were supposed to sample 35 students from each school. Each student of age 15 in each classroom had a real probability of participation (OECD, 2016).

^5^Students were asked to report the extent to which they agree with the statements: “My life has clear meaning or purpose”; “I have discovered a satisfactory meaning in life”; and “I have a clear sense of what gives meaning to my life.”

^6^Supplementary Table S2 presents mean values for all five well-being outcomes by country.

^7^It should be noted that each time a peer average is constructed in this study the focus individual was excluded from the calculation to avoid reverse causality.

^8^This is the regression sample that will be used for the life satisfaction regression. The samples for other well-being outcomes may slightly differ. The focus of this paper’s analysis will be on life satisfaction.

^9^The CEA region includes the following countries/economies: B-S-J-Z (China), Chinese Taipei, Hong Kong, Japan, South Korea, and Macao. Not all variables were available for Vietnam and Singapore. For China, PISA 2018 was conducted in the following four high-income provinces/municipalities: Beijing, Shanghai, Jiangsu and Zhejiang.

^10^*E(LS)* denotes the expected value of life satisfaction, *X* is a vector containing all predictors and a constant, and *β* contains the slope parameters and the intercept.

^11^I started out with a broader set of variables first which resulted in a significant loss in observations. Subsequently, I dropped the variables that were not regularly significant, increasing the sample size. There is usually a tradeoff involved in survey data in that model parsimony results in a larger sample size and vice versa.

^12^Given the nature of PISA data, high-income and middle-income regions are covered. High-income regions include four European regions, the United States, and Confucian East Asia. Middle-income regions include non-Confucian East Asia, Latin America, Middle East and North Africa, and the CIS region. For the country composition of each region see Supplementary Table S2.

^13^The Commonwealth of Independent States (CIS) comprises former Soviet Union countries.

^14^Estimations for the other four well-being measures produce similar results, but are not presented here for space reasons. They are, however, available from the author upon request.

## References

[ref1] Altonji J. G. Mansfield R. K. (2014). Group-average observables as controls for sorting on unobservables when estimating group treatment effects: The case of school and neighborhood effects (Working Paper No. 20781). Cambridge, MA: National Bureau of Economic Research.

[ref2] Antaramian S. P. Huebner E. S. Valois R. F. (2008). Adolescent life satisfaction. Appl. Psychol. 57, 112–126. doi: 10.1111/j.1464-0597.2008.00357.x

[ref3] Bandura A. (1982). Self-efficacy mechanism in human agency. Am. Psychol. 37, 122–147. doi: 10.1037/0003-066X.37.2.122

[ref4] Bastian B. Kuppens P. De Roover K. Diener E. (2014). Is valuing positive emotion associated with life satisfaction? Emotion 14, 639–645. doi: 10.1037/a0036466, PMID: 24749643

[ref5] Bergman M. M. Scott J. (2001). Young adolescents’ wellbeing and health-risk behaviours: gender and socio-economic differences. J. Adolesc. 24, 183–197. doi: 10.1006/jado.2001.0378, PMID: 11437479

[ref6] Blinder A. S. (1973). Wage discrimination: reduced form and structural estimates. J. Hum. Resour. 8, 436–455. doi: 10.2307/144855

[ref7] Bradshaw J. Martorano B. Natali L. De Neubourg C. (2013). Children’s subjective well-being in rich countries. Child Indic. Res. 6, 619–635. doi: 10.1007/s12187-013-9196-4

[ref8] Casas F. Rees G. (2015). Measures of children’s subjective well-being: analysis of the potential for cross-national comparisons. Child Indic. Res. 8, 49–69. doi: 10.1007/s12187-014-9293-z

[ref9] Chen B. Vansteenkiste M. Beyers W. Boone L. Deci E. L. Van der Kaap-Deeder J.. (2015). Basic psychological need satisfaction, need frustration, and need strength across four cultures. Motiv. Emot. 39, 216–236. doi: 10.1007/s11031-014-9450-1

[ref10] Chiu M. M. Chow B. W. Y. McBride C. Mol S. T. (2016). Students’ sense of belonging at school in 41 countries: cross-cultural variability. J. Cross-Cult. Psychol. 47, 175–196. doi: 10.1177/0022022115617031

[ref11] Clark A. E. Frijters P. Shields M. A. (2008). Relative income, happiness, and utility: an explanation for the Easterlin paradox and other puzzles. J. Econ. Lit. 46, 95–144. doi: 10.1257/jel.46.1.95

[ref12] Clark A. E. Oswald A. J. (1996). Satisfaction and comparison income. J. Public Econ. 61, 359–381. doi: 10.1016/0047-2727(95)01564-7

[ref13] Coffey J. K. Warren M. T. Gottfried A. W. (2015). Does infant happiness forecast adult life satisfaction? Examining subjective well-being in the first quarter century of life. J. Happiness Stud. 16, 1401–1421. doi: 10.1007/s10902-014-9556-x

[ref14] Conzo P. Aassve A. Fuochi G. Mencarini L. (2017). The cultural foundations of happiness. J. Econ. Psychol. 62, 268–283. doi: 10.1016/j.joep.2017.08.001

[ref15] Coupe T. Obrizan M. (2018). Adolescents’(un) happiness in transition. J. Comp. Econ. 46, 858–873. doi: 10.1016/j.jce.2018.07.012

[ref16] Deaton A. (2008). Income, health, and well-being around the world: evidence from the Gallup world poll. J. Econ. Perspect. 22, 53–72. doi: 10.1257/jep.22.2.53, PMID: 19436768 PMC2680297

[ref17] Diener E. (1994). Assessing subjective well-being: Progress and opportunities. Soc. Indic. Res. 31, 103–157. doi: 10.1007/BF01207052

[ref18] Diener E. Biswas-Diener R. (2002). Will money increase subjective well-being? Soc. Indic. Res. 57, 119–169. doi: 10.1023/A:1014411319119

[ref19] Diener E. Chan M. Y. (2011). Happy people live longer: subjective well-being contributes to health and longevity. Appl. Psychol. Health Well Being 3, 1–43. doi: 10.1111/j.1758-0854.2010.01045.x

[ref20] Diener E. Diener M. (1995). Cross-cultural correlates of life satisfaction and self-esteem. J. Pers. Soc. Psychol. 68, 653–663. doi: 10.1037/0022-3514.68.4.653, PMID: 7738768

[ref21] Diener E. Lucas R. E. (1999). 11 Personality and Subjective well-Being. Well-Being: Foundations of Hedonic Psychology. London: Sage, 213.

[ref22] Diener E. Suh E. M. (2000). Culture and subjective well-being. Cambridge, MA: MIT Press.

[ref23] Ferrer-i-Carbonell A. Frijters P. (2004). How important is methodology for the estimates of the determinants of happiness? Econ. J. 114, 641–659. doi: 10.1111/j.1468-0297.2004.00235.x

[ref24] Fredrickson B. L. (2001). The role of positive emotions in positive psychology: the broaden-and-build theory of positive emotions. Am. Psychol. 56, 218–226. doi: 10.1037/0003-066X.56.3.218 11315248 PMC3122271

[ref25] Fulmer C. A. Gelfand M. J. Kruglanski A. W. Kim-Prieto C. Diener E. Pierro A.. (2010). On “feeling right” in cultural contexts: how person-culture match affects self-esteem and subjective well-being. Psychol. Sci. 21, 1563–1569. doi: 10.1177/0956797610384742, PMID: 20876880

[ref26] Fwu B. J. Wang H. H. Chen S. W. Wei C. F. (2017). ‘Feeling bad’ or ‘being bad?’ The trapping effect of effort in academic failure in a Confucian cultural context. Educ. Psychol. 37, 506–519. doi: 10.1080/01443410.2016.1152355

[ref27] Gilman R. Huebner S. (2003). A review of life satisfaction research with children and adolescents. Sch. Psychol. Q. 18, 192–205. doi: 10.1521/scpq.18.2.192.21858

[ref28] Gilman R. Huebner E. S. (2006). Characteristics of adolescents who report very high life satisfaction. J. Youth Adolesc. 35, 293–301. doi: 10.1007/s10964-006-9036-7

[ref29] Helliwell J. F. Layard R. Sachs J. D. (2018). World happiness report. Gallup, Oxford Wellbeing Research Centre, UN Sustainable Development Solutions Network.

[ref30] Inglehart R. Foa R. Peterson C. Welzel C. (2008). Development, freedom, and rising happiness: a global perspective (1981–2007). Perspect. Psychol. Sci. 3, 264–285. doi: 10.1111/j.1745-6924.2008.00078.x, PMID: 26158947

[ref31] Jann B. (2008). The Blinder–Oaxaca decomposition for linear regression models. Stata J. 8, 453–479. doi: 10.1177/1536867X0800800401

[ref32] Joshanloo M. Lepshokova Z. K. Panyusheva T. Natalia A. Poon W. C. Yeung V. W. L.. (2014). Cross-cultural validation of fear of happiness scale across 14 national groups. J. Cross-Cult. Psychol. 45, 246–264. doi: 10.1177/0022022113505357

[ref33] Lee B. J. Yoo M. S. (2015). Family, school, and community correlates of children’s subjective well-being: an international comparative study. Child Indic. Res. 8, 151–175. doi: 10.1007/s12187-014-9285-z

[ref34] Lemma P. Borraccino A. Berchialla P. Dalmasso P. Charrier L. Vieno A.. (2015). Well-being in 15-year-old adolescents: a matter of relationship with school. J. Public Health 37, 573–580. doi: 10.1093/pubmed/fdu095, PMID: 25525193 PMC4659446

[ref35] Levin K. A. Currie C. (2010). Family structure, mother-child communication, father-child communication, and adolescent life satisfaction. Health Educ. 110, 152–168. doi: 10.1108/09654281011038831

[ref36] Lewis A. D. Huebner E. S. Malone P. S. Valois R. F. (2011). Life satisfaction and student engagement in adolescents. J. Youth Adolesc. 40, 249–262. doi: 10.1007/s10964-010-9517-6 20204687

[ref37] Li M. Fan W. Chen X. Cheung F. M. (2019). Independent and interdependent personalities at individual and group levels: predicting loneliness in Chinese adolescents. Personal. Individ. Differ. 147, 85–90. doi: 10.1016/j.paid.2019.04.031

[ref38] Lundqvist L. O. Dimberg U. (1995). Facial expressions are contagious. J. Psychophysiol. 9:203.

[ref39] Mahmoud J. S. R. Staten R. T. Hall L. A. Lennie T. A. (2012). The relationship among young adult college students’ depression, anxiety, stress, demographics, life satisfaction, and coping styles. Issues Ment. Health Nurs. 33, 149–156. doi: 10.3109/01612840.2011.632708, PMID: 22364426

[ref40] Marquez J. Main G. (2020). Can schools and education policy make children happier? A comparative study in 33 countries. Child Indic. Res. 12, 1–57. doi: 10.1007/s12187-020-09758-0

[ref41] Moksnes U. K. Løhre A. Lillefjell M. Byrne D. G. Haugan G. (2016). The association between school stress, life satisfaction and depressive symptoms in adolescents: life satisfaction as a potential mediator. Soc. Indic. Res. 125, 339–357. doi: 10.1007/s11205-014-0842-0

[ref42] Morgan A. R. Rivera F. Moreno C. Haglund B. J. (2012). Does social capital travel? Influences on the life satisfaction of young people living in England and Spain. BMC Public Health 12:138. doi: 10.1186/1471-2458-12-138, PMID: 22353283 PMC3311094

[ref43] OECD (2013). OECD guidelines on measuring subjective well-being. Paris: OECD Publishing. 24600748

[ref44] OECD (2016). PISA 2018 integrated design. Paris: OECD Publishing.

[ref45] OECD. (2019), PISA 2018 results (volume I): what students know and can do. OECD Publishing, Paris.

[ref46] Oishi S. Diener E. (2009). “Goals, culture, and subjective well-being” in Culture and well-being. ed. Diener E. (Dordrecht: Springer), 93–108.

[ref47] Park N. (2004). The role of subjective well-being in positive youth development. Ann. Am. Acad. Pol. Soc. Sci. 591, 25–39. doi: 10.1177/0002716203260078

[ref48] Park N. Huebner E. S. (2005). A cross-cultural study of the levels and correlates of life satisfaction among adolescents. J. Cross-Cult. Psychol. 36, 444–456. doi: 10.1177/0022022105275961

[ref49] Raboteg-Saric Z. Sakic M. (2014). Relations of parenting styles and friendship quality to self-esteem, life satisfaction and happiness in adolescents. Appl. Res. Qual. Life 9, 749–765. doi: 10.1007/s11482-013-9268-0

[ref50] Rudolf R. (2020). Changing paradigms in measuring national well-being: how does Korea rank “beyond GDP”? Asian Soc. Work Policy Rev. 14, 118–121. doi: 10.1111/aswp.12195

[ref51] Rudolf R. Bethmann D. (2023). The paradox of wealthy nations’ low adolescent life satisfaction. J. Happiness Stud. 24, 79–105. doi: 10.1007/s10902-022-00595-2

[ref52] Rudolf R. Kang S. J. (2015). Lags and leads in life satisfaction in Korea: when gender matters. Fem. Econ. 21, 136–163. doi: 10.1080/13545701.2014.967708

[ref53] Rudolf R. Lee J. (2023). School climate, academic performance, and adolescent well-being in Korea: the roles of competition and cooperation. Child Indic. Res. 16, 917–940. doi: 10.1007/s12187-022-10005-x

[ref54] Ryan R. M. Deci E. L. (2000). Self-determination theory and the facilitation of intrinsic motivation, social development, and well-being. Am. Psychol. 55, 68–78. doi: 10.1037/0003-066X.55.1.68, PMID: 11392867

[ref55] Ryan R. M. Deci E. L. (2017). Self-determination theory: basic psychological needs in motivation, development, and wellness. New York, NY: Guilford Publications.

[ref56] Senik C. (2014). The French unhappiness puzzle: the cultural dimension of happiness. J. Econ. Behav. Organ. 106, 379–401. doi: 10.1016/j.jebo.2014.05.010

[ref57] Shek D. T. Li X. (2016). Perceived school performance, life satisfaction, and hopelessness: a 4-year longitudinal study of adolescents in Hong Kong. Soc. Indic. Res. 126, 921–934. doi: 10.1007/s11205-015-0904-y 26912946 PMC4751166

[ref58] Stankov L. (2010). Unforgiving Confucian culture: a breeding ground for high academic achievement, test anxiety and self-doubt? Learn. Individ. Differ. 20, 555–563. doi: 10.1016/j.lindif.2010.05.003

[ref59] Steel P. Schmidt J. Shultz J. (2008). Refining the relationship between personality and subjective well-being. Psychol. Bull. 134, 138–161. doi: 10.1037/0033-2909.134.1.138 18193998

[ref60] Steger M. F. Kashdan T. B. Sullivan B. A. Lorentz D. (2008). Understanding the search for meaning in life: personality, cognitive style, and the dynamic between seeking and experiencing meaning. J. Pers. 76, 199–228. doi: 10.1111/j.1467-6494.2007.00484.x, PMID: 18331281

[ref61] Stutzer A. Frey B. S. (2006). Does marriage make people happy, or do happy people get married? J. Socio-Econ. 35, 326–347. doi: 10.1016/j.socec.2005.11.043

[ref62] Suh E. M. Choi S. (2018). “Predictors of subjective well-being across cultures” in Handbook of well-being. eds. Diener E. Oishi S. Tay L. (Salt Lake City, UT: DEF Publishers).

[ref63] Suh E. Diener E. Oishi S. Triandis H. C. (1998). The shifting basis of life satisfaction judgments across cultures: emotions versus norms. J. Pers. Soc. Psychol. 74, 482–493. doi: 10.1037/0022-3514.74.2.482

[ref64] Suldo S. M. Huebner E. S. (2004). Does life satisfaction moderate the effects of stressful life events on psychopathological behavior during adolescence? Sch. Psychol. Q. 19, 93–105. doi: 10.1521/scpq.19.2.93.33313

[ref65] Sun R. C. Shek D. T. (2013). Longitudinal influences of positive youth development and life satisfaction on problem behaviour among adolescents in Hong Kong. Soc. Indic. Res. 114, 1171–1197. doi: 10.1007/s11205-012-0196-4

[ref66] Tong Y. Song S. (2004). A study on general self-efficacy and subjective well-being of low SES-college students in a Chinese university. Coll. Stud. J. 38, 637–643.

[ref67] Tsai W. Lau A. S. (2013). Cultural differences in emotion regulation during self-reflection on negative personal experiences. Cognit. Emot. 27, 416–429. doi: 10.1080/02699931.2012.715080, PMID: 22916683

[ref68] Tumen S. Zeydanli T. (2015). Is happiness contagious? Separating spillover externalities from the group-level social context. J. Happiness Stud. 16, 719–744. doi: 10.1007/s10902-014-9531-6

[ref69] Uchida Y. Kitayama S. Mesquita B. Reyes J. A. S. Morling B. (2008). Is perceived emotional support beneficial? Well-being and health in independent and interdependent cultures. Personal. Soc. Psychol. Bull. 34, 741–754. doi: 10.1177/0146167208315157, PMID: 18359927

[ref70] Uchida Y. Ogihara Y. (2012). Personal or interpersonal construal of happiness: a cultural psychological perspective. Int. J. Wellbeing 2, 354–369. doi: 10.5502/ijw.v2.i4.5

[ref71] Yang Y. Li P. Kou Y. (2017). Orientations to happiness and subjective well-being in Chinese adolescents. Child Indic. Res. 10, 881–897. doi: 10.1007/s12187-016-9410-2

[ref72] Zelenski J. M. Murphy S. A. Jenkins D. A. (2008). The happy-productive worker thesis revisited. J. Happiness Stud. 9, 521–537. doi: 10.1007/s10902-008-9087-4

