# Supplementary material for: Predictors of adolescent well-being around the globe: are students from Confucian East Asia different?
Source: Front Psychol. 2024 Oct 28;15:1446301. doi: 10.3389/fpsyg.2024.1446301 (PMC11551778; doi:10.3389/fpsyg.2024.1446301)
Supplement: Supplementary file 1 [file Data_Sheet_1.pdf]

Electronic Supplementary Material

Table S1: Description of variables

Variable	Description	Response scale
Subjective well-being		
Life satisfaction	Overall, how satisfied are you with your life as a whole these days?	from "0" = 'not at all satisfied' to "10" = 'completely satisfied'
Happiness	Thinking about yourself and how you normally feel: how often do you feel happy?	"1" = 'never', "2" = 'rarely', "3" = 'sometimes', "4" = 'always'
Positive affect	Average of emotions happy, joyful, cheerful, lively. Measurement analogous to happiness above.	
Negative affect	Average of emotions afraid, scared, sad, miserable. Measurement analogous to happiness above.	
Meaning in life index	PISA 2018 asked students (ST185) to report the extent to which they agree (“strongly agree”, “agree”, “disagree”, “strongly disagree”) with the following statements: “My life has clear meaning or purpose”; “I have discovered a satisfactory meaning in life”; and “I have a clear sense of what gives meaning to my life”. These three statements were combined to form the index of meaning in life (EUDMO). Positive values in the index indicate greater meaning in life than the average student across OECD countries.	
Individual characteristics		
Female	Gender of student	"1" = female, "0" = male
Single child	Absence of siblings	"1" = student is the only child; "0" otherwise
Migrant background	Indication of migrant background in family	"1" = student is first or second generation migrant; "0" otherwise

Self-efficacy/ resilience index	PISA 2018 asked (ST188) students to report the extent to which they agree (“strongly disagree”, “disagree, “agree”, “strongly agree”) with the following statements about themselves: “I usually manage one way or another”; “I feel proud that I have accomplished things”; “I feel that I can handle many things at a time”; “My belief in myself gets me through hard times”; and “When I’m in a difficult situation, I can usually find my way out of it”. These statements were combined to create the index of self-efficacy (RESILIENCE). Positive values in this index mean that the student reported higher self-efficacy than did the average student across OECD countries.	
Emotional interdependence	Level of agreement to the statement "When I am failing, I worry about what others think of me."	"1" = 'strongly disagree', "2" = 'disagree', "3" = 'agree', "4" = 'strongly agree'
Family characteristics		
Parents max years of schooling	Highest education level of parents.	years of schooling
Home possessions index	In PISA 2018, students reported the availability of 16 household items at home (ST011), including three country-specific household items that were seen as appropriate measures of family wealth within the country’s context. In addition, students reported the amount of possessions and books at home (ST012, ST013). HOMEPOS is a summary index of all household and possession items (ST011, ST012 and ST013).	
Parents' emotional support index	The index of parents’ emotional support (EMOSUPS) was constructed based on a trend question (ST123) asking students whether they agree (“strongly agree”, “agree”, “disagree”, “strongly disagree”) with the following statements: “My parents support my educational efforts and achievements”; “My parents support me when I am facing difficulties at school”; and “My parents encourage me to be confident”. Positive values on this scale mean that students perceived greater levels of emotional support from their parents than did the average student across OECD countries.	
Autonomy/ learning environment at home		
Own room	Whether student has an own room at home.	"1" = yes; "0" = no
Desk to study	Whether student has a desk to study at home.	"1" = yes; "0" = no
Quiet place to study	Whether student has a quiet place to study at home.	"1" = yes; "0" = no
Peer characteristics		
Life satisfaction (peer avg.)	Average of school peers' life satisfaction. Focus individual is excluded.	
Happiness (peer avg.)	Average of school peers' happiness. Focus individual is excluded.	
Positive affect (peer avg.)	Average of school peers' positive affect. Focus individual is excluded.	

Negative affect (peer avg.)	Average of school peers' negative affect. Focus individual is excluded.
Meaning in life (peer avg.)	Average of school peers' perceived meaning in life. Focus individual is excluded.
Home possessions index (peer avg.)	Average of school peers' home possessions. Focus individual is excluded.
Parents max years of schooling (peer avg.)	Average of school peers' parents' max years of schooling. Focus individual is excluded.
School atmosphere	
Student competition index (school avg.)	PISA 2018 asked (ST205) students how true ("not at all true", "slightly true", "very true", "extremely true") the following statements about their school are: "Students seem to value competition"; "It seems that students are competing with each other"; "Students seem to share the feeling that competing with each other is important"; and "Students feel that they are being compared with others". The first three statements were combined to create the index of student competition (PERCOMP). Positive values in this index mean that students perceived their peers to compete with each other to a greater extent than did the average student across OECD countries. The index was averaged over all participating students in an adolescent's school.
Student cooperation index (school avg.)	PISA 2018 asked (ST206) students how true ("not at all true", "slightly true", "very true", "extremely true") the following statements about their school are: "Students seem to value co-operation"; "It seems that students are co-operating with each other"; "Students seem to share the feeling that co-operating with each other is important"; and "Students feel that they are encouraged to cooperate with others". The first three statements were combined to create the index of student co-operation (PERCOOP). Positive values in this index mean that students perceived their peers to co-operate to a greater extent than did the average student across OECD countries. The index was averaged over all participating students in an adolescent's school.
Teacher support index (school avg.)	The index of teacher support (TEACHSUP) was constructed using students' responses to a trend question (ST100) about how often ("every lesson", "most lessons", "some lessons", "never or hardly ever") the following things happen in their language-of-instruction lessons: "The teacher shows an interest in every student's learning"; "The teacher gives extra help when students need it"; "The teacher helps students with their learning"; and "The teacher continues teaching until the students understand". Positive values on this scale mean that students perceived their teacher to be more supportive than did the average student across OECD countries. The index was averaged over all participating students in an adolescent's school.
Sense of belonging index	The index of sense of belonging (BELONG) was constructed using students' responses to a trend question about their sense of belonging to school. Students were asked whether they agree ("strongly agree", "agree", "disagree", "strongly disagree") with the following statements (ST034): "I feel like an outsider (or left out of things) at school"; "I make friends easily at school"; "I feel like I belong at school"; "I feel awkward and out of place in my school"; "Other students seem to like me"; and "I feel lonely at school". Three of these items were reversed-coded so that positive values on this scale mean that students reported a greater sense of belonging at school than did the average student across OECD countries.
Level of urbanization	

Village	Student lives in a village.	"1" = yes; "0" = no
Small town	Student lives in a small town.	"1" = yes; "0" = no
Town	Student lives in a town.	"1" = yes; "0" = no
City	Student lives in a city.	"1" = yes; "0" = no
Large city	Student lives in a large city.	"1" = yes; "0" = no

Table S2: Adolescent life satisfaction by country

World region	Country/ Economy	Mean	SD
Western Europe	Austria	7.14	5.63
	France	7.19	1.45
	Germany	7.02	1.59
	Luxembourg	7.04	17.41
	Netherlands	7.50	1.89
	Switzerland	7.38	4.63
Eastern Europe	Bulgaria	7.15	6.90
	Czech Republic	6.91	5.40
	Hungary	7.12	4.38
	Moldova	7.68	7.87
	Poland	6.74	2.52
	Romania	7.87	3.08
	Slovak Republic	7.22	6.83
	Ukraine	8.03	2.17
Northern Europe	Estonia	7.19	11.75
	Finland	7.61	4.96
	Iceland	7.34	15.64

	Ireland	6.74	5.40
	Latvia	7.16	9.82
	Lithuania	7.61	9.19
	Sweden	7.01	4.42
	United Kingdom	6.16	2.91
Southern Europe	Albania	8.61	7.11
	Bosnia and Herzegovina	7.84	8.63
	Croatia	7.69	7.57
	Greece	6.99	4.60
	Italy	6.91	2.61
	Kosovo	8.30	7.61
	Malta	6.56	17.46
	Montenegro	7.69	19.70
	North Macedonia	8.16	9.35
	Portugal	7.13	3.90
	Serbia	7.61	6.18
	Slovenia	6.86	11.92
	Spain	7.35	4.88
United States	United States	6.75	0.68
Latin America	Argentina	7.26	2.85
	Brazil	7.05	1.49
	Chile	7.03	3.70
	Colombia	7.62	2.29
	Costa Rica	7.96	6.57
	Dominican Republic	8.09	3.90
	Mexico	8.11	1.13
	Panama	7.92	7.32
	Peru	7.31	2.18

	Uruguay	7.54	6.56
Confucian East Asia	Taiwan/ Chinese Taipei	6.52	3.07
	Hong Kong	6.27	5.55
	Japan	6.18	1.40
	Korea	6.52	2.27
	Macao	6.07	16.73
	Vietnam	7.47	1.15
	B-S-J-Z (China)	6.64	1.99
Non-Confucian East Asia	Brunei Darussalam	5.80	17.15
	Indonesia	7.47	1.05
	Malaysia	7.04	2.17
	Philippines	7.21	1.35
	Thailand	7.64	1.98
Middle East & North Africa (MENA)	Jordan	6.88	6.42
	Lebanon	6.67	6.31
	Morocco	6.95	2.90
	Qatar	6.84	19.78
	Saudi Arabia	7.95	2.51
	Turkey	5.62	1.93
	United Arab Emirates	6.88	12.02
Commonwealth of Independent States (CIS)	Baku (Azerbaijan)	7.24	12.97
	Belarus	8.10	3.99
	Georgia	7.60	7.26
	Kazakhstan	8.76	4.44
	Russian Federation	7.32	1.50
	Moscow Region (RUS)	7.27	3.51
	Tatarstan (RUS)	7.55	7.69

Notes: Summary stats calculated using population weights. World region classification according to UN Statistics.
